# Pathogenesis, Clinical Features, Diagnosis, and Management of Radiation Hazards in Dentistry

**DOI:** 10.2174/1745017901814010742

**Published:** 2018-09-28

**Authors:** Gundeep Singh, Amit Sood, Ambreen Kaur, Deepak Gupta

**Affiliations:** 1 Department of Pedodontics, Haldia College of Dental Sciences and Research, West Bengal, India; 2 Department of Conservative Dentistry and Endodontics, Shaheed Kartar Singh Sarabha Dental College and Hospital, Ludhiana, Punjab, India; 3 Department of Orthodontics, Luxmi Bai Dental College, Patiala, Punjab, India; 4 Department of Oral Medicine and Radiology, M.M.College of Dental Sciences and Research, Mullana, Ambala, Haryana, India

**Keywords:** Radiation induced effects, Management, Radiation, Dental professionals, Carcinogenesis, Radiation safety

## Abstract

**Introduction:**

With the advent of newer radiographic diagnostic procedures of the maxillofacial region, there is a drastic increase in the use of Ionizing radiation which further leads to increased chance of radiation hazards among the patients and the health care workers. In addition to the diagnostic information extracted, the radiation exposure carries the potential to induce carcinogenesis in the exposed individual. However, the amount of Radiation exposure in dentistry is significantly low but it is still harmful owing to the requirement of repeated radiographic examination during the dental treatment. Therefore, to ensure minimum and inevitable exposure during dental treatment, it is necessary to follow principles of radiation protection and safety.

**Recommendations:**

Several studies in the literature have revealed that the attitude and knowledge of the dental professionals regarding radiation safety is not up to the mark. Henceforth, there is a necessity of implementing certain basic guidelines regarding radiation safety and protection. Further state dental councils must advocate new and interesting methods of education regarding the same and should introduce strict rules and penalties for this spectrum of field.

**Conclusion:**

This present short commentary is to familiarize the dental practitioner regarding the methods to minimize the risk of the radiation hazards. Further this article will also educate the dental practitioners regarding the pathogenesis of Radiation effects during Radiation therapy of head and neck region along with pertinent management protocols.

## 1 INTRODUCTION

Ionizing radiation is defined as a radiation which has sufficient energy to ionize biological molecules [1]. Exposure to such radiation for human tissue is harmful. X-rays which are widely used in diagnostic radiology in medical as well as dental fields are also a type of Ionizing radiation [2]. Henceforth the patients get exposed to this ionizing radiation while obtaining radiographs resulting in the modification of biological macromolecules. The literature reveals that even one single exposure of the patient to intraoral periapical radiograph is capable ofcausing genotoxic effects to the exposed cells of the individual [1].

The biologic effects of radiation are divided into two varieties. The first are stochastic effects in which the probability of the occurrence of an effect rather than severity is proportional to the dose. These effects include cancer, mutation and effects on the embryo. They follow the principle of all or none and do not have any dose thresholds [3-6].

It is a well known fact that dental professionals are most frequent with performing X-ray investigation as compared to medical practitioners [7]. Almost all the dental treatments like RCT’s, extractions, implants *etc* require radiographs for proper treatment planning. Further, the literature also reveals high paediatric radiological investigations too. Most often radiological procedure performed is an Intra oral periapical radiograph which is done to evaluate the tooth and its periapical area. It is well documented by several researchers that the radiation exposure during dental radiograph such as IOPA as well as OPG is quite low [8, 9] however unwanted and repeated examinations must be avoided [10, 11].

It is widely observed that there is a lack in the quality assurance programmes as far as the radiographs are concerned [12]. Hence, these radiation safety measures are considered important for the dental professionals. The exposure to radiation in the maxillofacial region may attribute to the tumors of salivary glands, cancer of thyroid gland and meningioma to name a few [12]. Further it may also lead to low birth weight in the new borns of exposed pregnant females [12]. Henceforth, justification and optimization of dental radiology is considered an important aspect for dental professionals so as to reduce the unwanted radiation exposure [7, 8]. This in turn necessitates the inclusion of radiation protection protocols during the practice of radiologic examination [13-17].

Several countries have international and national organizations which are considered responsible for data analysis and publication of reports concerned to information on radiation protection [1, 13-17].

Few of these international level organizations are:

- International Commission of Radiological Protection (ICRP),
- United Nations Scientific Committee on the Effects of Atomic Radiation (UNSCEAR) and
- International Commission on Radiation Units and Measurements (ICRU) [1].

It is of interest to know that United Nations Scientific Committee on Effects of Atomic Radiation (UNSCEAR) gave a report regarding diagnostic radiography in dentistry. The committee reported that almost 480 million radiographic examinations were performed in the whole world in 2008 specifically for dental diagnosis [6]. This constitutes of 15% of the annual diagnostic X-ray examinations in health care [6].

Apart of the fact that the dental professionals know about the harmful nature of X-radiation, still the literature reveals their casual attitude while performing dental radiographs. Many authors worldwide have conducted their research on radiation safety. On the basis of these studies, strict radiation safety guidelines have to be followed so as to achieve the safety of dental patient as well as the dental professional. All the dental professionals must follow the guiding principles for radiation protection [12].

Further, the knowledge of dental practitioners regarding the management of patients undergoing radiation therapy for maxillofacial region is not up to date. The dental professionals must be competent enough to manage such patients.

## 2 ALARA

ALARA stands for As Low As Reasonable Achievable. It is considered as a guiding principle in radiation protection [6, 12, 13]. According to this principle, the Dental professionals are ethically obligated towards minimizing exposure and hence maximizing the diagnostic result for the patient. Further, the dentist must apply selection criteria while prescribing radiographs to the patients and the radiographic examinations must be performed with the recommended safety measures.

The dental professional must emphasize upon optimal imaging, exposure and processing techniques so as to prevent re-exposure of the patient [14, 15].

### 2.1 Minimizing Exposure of the Patient

Following are the measures that can be followed in adherence to ALARA principle so as to reduce the exposure of the dental patient as well as the operator.

#### 2.1.1 Standard X-ray Techniques

The dental professionals must use standard X ray techniques with accuracy. This will provide with a good quality radiograph thus reducing re-exposure of the patient.

#### 2.1.2 Selective Peri-apical Views

The dental professionals must give preference to the selective peri-apical view to the patients in their initial visits [6, 7].

#### 2.1.3 Periodic Evaluation of X-Ray Equipment

This is required so as to assure appropriate exposure by X-radiation. Further this also gives a clue if there is any radiation leakage [6, 16]. Literature reveals that the dentists are not habitual for the periodic evaluation of their X-ray equipment in many parts of the world. This leads unnecessary radiation exposure due to the radiation leakage [16]. A well calibrated dental X-ray machine according to Praveen BN must possess and output of 0.7 to 1R/sec [17]. Further, this calibration must be assessed after every 3 years [17].

#### 2.1.4 Paralleling Angle Technique

It is of interest to know that a part of the fact that paralleling technique involves less exposure, still Bisecting angle technique is more common for taking intra oral periapical radiographs [18-20]. Bisecting angle technique involves steep vertical angulation which provides more radiation exposure to the thyroid gland as well as the eye lens [17].

#### 2.1.5 Image Receptors and Speed of Film

 Earlier it was Radiographic film only which was widely used as image receptors. However, these days the conventional film receptors are greatly replaced by digital receptors. Various digital receptors include Charged-Coupled Device (CCD) receptors, the Complementary Metal Oxide Semiconductor (CMOS) and Photostimulable Phosphor Plate (PSP) receptors.

The speed of radiographic film is dependent upon the sensitivity of the emulsion of the film to x-rays. Hence less radiation exposure will be required for fast films and they will be considered more beneficial to the patient.

Several films like D, E, and F speed films are commercially available. Film F is the fastest speed film. Further it is also ascertained that the use of F speed films can significantly decrease the exposure to the patient by 70% when compared to D speed films and 20% when compared to E speed films that too without affecting the image quality [21]. Keeping this fact in mind, the FDA has recommended not to use any film below E speed [21].

It is also important to note that digital radiography (radiovisuography) requires less radiation exposure as compared to conventional intraoral periapical films. It is known to reduce the patient exposure by 75% when compared to D speed films, 50% when compared to E speed and 40% when compared to F speed films [22].

#### 2.1.6 Intensifying Screens

Intensifying screens: Rare earth intensifying screens can help to reduce radiation exposure in film based extraoral radiography like panoramic radiographs and cephalometric radiographs by 55% [23]. Rare earth phosphors emit a green light upon X-ray exposure. Hence when green light sensitive films are combined with rare earth screens, it can result in the reduction of Exposure. Digital receptors in the form of PSP plates can be used for extraoral radiography in panoramic and cephalometric film cassettes. On the contrary literature also reveals no significant reduction in dose when digital receptors are used in place of rare-earth intensifying screens that too in combination with matched high-speed extraoral film [24].

#### 2.1.7 Collimation

Collimators refers to restriction of the X-ray beam size which results in reducing patient’s exposure. Intraoral radiographs utilizes two types of collimators *i.e.* round and rectangular collimators. Rectangular collimators are considered to be better as these expose 60% less tissue as compared to round collimators. Therefore rectangular collimators are recommended with X-ray beam [25, 26]. On the contrary more precision is required by the operator to use rectangular collimators in terms of receptor placement, angulations and beam alignment. It is recommended to exercise a strict clinical training if the clinician selects to use rectangular collimation [27, 28]. Further, the long Position Indicating Device (PID) (16” or 40 cm) should be preferred as compared to short PID (8” or 20 cm) so as to reduce unwanted tissue exposure [26].

#### 2.1.8 Filtration

Filtration is a process which involves removal of photons which possess low energy from the X-ray beam which if not removed will be absorbed by the patient. Aluminium is one of the most commonly used metal for filtration. The dental X-ray machines which operate at < 70 kVp must have 1.5 mm thickness of aluminium while those machines with operating kVp ≥ 70 kVp must possess 2.5 mm of aluminium filtration.

#### 2.1.9 Kilovoltage

Literature has revealed that those X-ray machines which operate at kVp less than 60 results in higher patient radiation exposure. The Dental professionals must keep the kVp between 60 to 80 [26].

#### 2.1.10 Exposure Time

Most of dentists do not change the exposure time of their machines white capturing the radiographs owing to the fact that exposure time must be changed on the basis of location of the tooth as well as patient characteristics [18]. This also leads to unnecessary patient exposure.

#### 2.1.11 Eliminate Chemical Processing Errors

Poor image quality is also attributed to usage of processing solutions even after their shelf life or strength. Further improper chemical processing techniques can also require re-exposure of the patient leading to unnecessary radiation dose. Henceforth proper chemical processing guidelines must be followed and solution change must be done at regular intervals [26].

#### 2.1.12 Use of Protective Devices

Unwanted radiation exposure to the Dental professionals can be prevented with the use of protective devices like lead apron and thyroid collor. The person performing the X-rays can be protected with the use of protective lead shield and lead gloves [18].

##### 2.1.12.1 Patient Shields

###### a Thyroid Collar

The thyroid gland is considered to be the most sensitive organ for radiation induced tumors [26]. In intraoral radiography, the thyroid gland is bound to get exposed even with proper radiographic techniques. It is of interest to know that the exposure of thyroid gland in pregnant females may lead to low birth weight babies. Henceforth it is recommended to use thyroid collar while performing intraoral radiographs especially for children and pregnant patients as it is estimated to reduce the exposure by 50% to the gland [29].

###### b Lead Shields

Certain guidelines have recommend the use of lead apron while capturing radiographs of children, infants and pregnant females [29-31]. Praveen BS *et al*. revealed that unwanted radiation exposure to the developing fetus during 8 to 15 weeks of pregnancy may lead to greatest risk for chrosomal abnormalities and even mental retardation [9].It is strongly recommended that the personnel exposing the patient during dental radiographs must stand behind lead shield. Several studies in the literature have confirmed that atleast 0.25 mm of lead or its equilant is required for effective shielding from X-radiation. NCRP even recommends annual inspection of lead shield for any leakage [26].

### 2.2 Minimizing Operator Exposure

The operator or the dental professional must practice standard safety measures to prevent occupational exposure [24, 26, 29, 30].

This includes:

- The dental assistant or operator must not stand in line of the primary beam.
- He/she should not support or hold the X-ray tube head or image receptors in the patient’s mouth.
- The dental X-ray operator must utilize a radiation barrier.
- In case barriers are not available, the distance and position rule must be kept in consideration. According to this rule, the operator must be atleast 2 meters (6 feet 8 inches) away from the X-ray source. Further the operator must stand between 90˚ - 135˚ angle to the primary X-ray beam [26, 29, 30].
- Barrier specifications may change according to the type of dental radio graphical equipment or workload. It may also depend upon factors such as the maximum kVp of the machine and distance from the X-ray source as well as radiation status of the people the facility.

According to NCRP’s recommendation, the dental personnel must be monitored if they receive or are expected to receive an annual effective dose in excess of 0.1 rem or 1 mSv. Personal dosimeters must also be provided for those pregnant females who are known to get occupationally exposed [26]. Literature reveals that the Minimum Permissible Dose for an occupationally exposed pregnant radiation worker is considered limited to 0.5 rem or 5 mSv during pregnancy. Further monthly monitoring must be done for such individuals so as to help them keep their exposure below this limit [26].

#### 2.2.1 Occupational Radiation Monitoring

The dental professional must perform radiation monitoring with the help of a film badge dosimeter, TLD monitor (thermoluminescent dosimeter) or an optically stimulated luminescence dosimeter to monitor the maximum permissible dose for the person performing radiographs. The dental professional can wear the dosimeter at the collar level or waist or chest level [26, 30].

### 2.3 Radiation Biology

It is defined and that portion of science that studies the effects of ionizing radiation on living organisms. Since the radiation used during the time of diagnostic radiology and radiation therapy is ionizing, we need to know its effects on the living organisms. The initial interaction between ionizing radiation and matter occurs at the level of the electron. This happens within the first 10-13 second post exposure. This interaction in the ensuing seconds to hours may results in the modification of biologic molecules.

These molecular changes further lead to changes in the cells and organisms. These changes or alteration in the organisms can manifest for few hours or days or even for decades [32-34]. These can even manifest in future generations too [32-34]**.** Injury or death of the exposed individual will happen in case enough cells are killed in the individual. On the contrary, if the cells are modified, it may result in carcinogenesis or other disorders in the future generations of the exposed individuals.

#### 2.3.1 The Biologically Damaging Effects of Ionizing Radiation are Classified into Three Main Categories [32-34]

##### 2.3.1.1 Somatic Deterministic Effects

These are defined as those damaging effects resulting from a specific high radiation dose. In these types of effects, the severity of effect is proportional to the dose. They are dose dependent and possess a dose threshold below which there will be no response. Examples include oral changes seen after radiation therapy, skin reddening and cataract formation [32-34].

##### 2.3.1 2. Somatic Stochastic Effects

These are the effects in which the probability of the occurrence of a change, rather than its severity, is dose dependent. Henceforth these effects follow the law of probability and are manifested at random. It can be rightly said that they are all- or- none. These can manifest by exposure to any radiation dosage. It is of interest to note that experimentally there is no such SAFE DOSE which cannot manifest the Stochastic effects [32-34]. In other words, even a radiation dose as small as a single IOPA has got the capability to induce such changes. Hence there is a dire need to check the unwanted usage of ionizing radiation [32]. Examples of such effects include leukemia, certain tumors and radiation induced cancer [34].

##### 2.3.1.3 Genetic Stochastic Effects

These are those effects which can lead to Mutations resulting from any sudden changes to a gene or chromosomes. These changes can be triggered by external factors, such as radiation or may even manifest spontaneously. Radiation to the reproductive organs may damage the DNA of the sperm or egg cells. It may result in a congenital abnormality in the offspring of the person irradiated [32-34].

Mechanism of Radiation Injury [32-34]

- **Direct Effect:** These are the effects which happens when the X ray photon or the secondary electrons directly ionize the biologic tissues.
- **Indirect Effect:** These are the effects in which water acts as a medium. The X-ray photons are first absorbed by the water in the body of the individual leading to ionized water molecules. This further leads to formation of free radicals which in turn interacts and produce changes in biologic tissues.

#### 2.3.2 Effects of Radiation on Biological Molecules [32-34]

##### 2.3.2.1 Nucleic Acid

Radiation can induce different type of changes in the DNA which includes:

- Breakage or cross linking of DNA strands.
- Change or loss of base pair of disruption of hydrogen bonds in between DNA strands.

The most important types of damage are single and double strand breakage. Disrepair of a DNA strand results in mutation and consequent biologic effect.

##### 2.3.2.2 Proteins

Irradiation of proteins in solution usually leads to Changes in their secondary and tertiary structures

## 3 SHORT TERM EFFECTS

These are the effects which are determined primarily by the sensitivity of its parenchymal cells [32-34]. When a moderate amount of radiation dose is given to continuously proliferating tissues like bone marrow and oral mucous membrane, the cells get killed by mitosis linked death and the tissues will demonstrate radiation induced hypoplasia. However the biological tissues which are comprised of cells that either never divide or divide rarely like nervous tissues or muscular tissueswill demonstrate little or no cell death with radiation. Hence these tissues will not show any radiation induced hypoplasia over short term.

## 4 LONG TERM EFFECT

The long term deterministic effects of radiation depend primarily on the extent of damage to the fine vasculature [32-34]. Irradiation of capillaries inside the body will lead to Swelling, Degeneration and consequent necrosis of the cells in the walls of these blood vessels. This will further lead to increase in the capillary permeability and initiate a slow progressive fibrosis around the vessels. As this fibrosis is increased, the inner lumen of the vessels will narrow down eventually causing obliteration of the lumen of the vessels. This subsequently impairs the transport of oxygen, nutrients and waste products and results in death of all cell types with resultant progressive fibroapathy of the irradiated tissues. The cells as a result will lose their function and the irradiated tissues will suffer from reduced resistance to infection and trauma [32-34].

## 5 RADIATION EFFECT ON ORAL TISSUES [32-34]

### 5.1 Oral Mucous Membrane (OMM)

OMM constitutes of a basal cell layer containing vegetative and differentiating intermitotic cells which are radiosensitive. Due to radiation, some of these cells die during or at the end of 2^nd^ week of therapy. Due to this fact the mucous membrane will become Red and Inflamed. This clinical condition is known as mucositis [32].

#### 5.1.1 As the Therapy Continues [32, 33]

- It begins to break down.
- Formation of a yellowish white pseudo membrane occurs.

#### 5.1.2 At the End of Therapy

- Mucositis is most severe.
- Discomfort is at a maximum.
- Food intake is painful and difficult.

### 5.2 Management

- Maintain Good oral hygiene so as to minimize the infection.
- Advocate topical anaesthetics during the meal times.
- Treatment of secondary yeast infection by Candida albicans requires treatment as it is one of the most common complication.

#### 5.2.1 After the Completion of Radiation Therapy

- It begins to heal rapidly.
- Mostly healing is complete by about 2 months.

#### 5.2.2 At Later Intervals (months to years)

It tends to become atrophic. It also becomes thin and avascular relatively owing to progressive obliteration of vascular lumens. These atrophic changes creates problem is the patients with denture as there may be oral ulcerations of the compromised tissue due to trauma from the hard dentures [32].

### 5.3 Taste Buds

Taste buds are sensitive to radiation. Therapeutic doses during the second to third week of radiotherapy can cause extensive degeneration of the normal histologic architecture of taste buds which leads to loss of acuity of taste [33].

### 5.4 Salivary Glands

The parenchymal component of the salivary glands is radiosensitive. Parotid gland is usually more radiosensitive as compared to submandibular or sublingual glands [32].

#### 5.4.1 Following Changes Happen in 1^st^ Few Weeks after Initiation of Radiotherapy [32-34]

- Marked and progressive loss of salivary secretion
- Extent of reduce flow is dose dependent and reaches essentially 0 at 60 Gy.
- Mouth becomes dry and tender.
- Swallowing is difficult and painful as the residual saliva loses its normal lubricating properties.
- The small volume of viscous saliva that is secreted usually has a PH value 1 unit below normal, which is enough to initiate decalcification of normal enamel.
- It’s buffering capacity falls as much as 44%.
- If some portions of the major salivary glands have been spared, dryness of the mouth usually subsides in 6 to 12 months due to compensatory hypertrophy of residual salivary gland tissue.
- Reduced salivary flow that persists beyond a year is unlikely to show significant recovery.

Histologically an acute inflammatory response may occur soon after the initiation of therapy, particularly involving the serous acini.

#### 5.4.2 After Irradiation

- The inflammatory response becomes more chronic
- The glands demonstrate progressive fibrosis
- Adiposis
- Loss of fine vasculature
- Concomitant parenchymal degeneration which accounts for the xerostomia.

### 5.5 Teeth

Irradiation with therapeutic doses can retard the growth of the teeth if the irradiation occur during their development stage. Irradiation during or before the calcification stage of the teeth can even destroy the tooth in its bud form. Irradiation after calcification may inhibit cellular differentiation, causing malformations and arresting general growth. Children receiving radiation therapy of the jaws may show defects in the permanent dentition which includes Retarded root development, Dwarfed teeth and Failure to form one or more teeth. Teeth irradiated during development may complete calcification and erupt prematurely [32-34]. Irradiation of teeth may have an effect on the root formation while it is of interest to note that the eruptive mechanism of teeth is relatively radiation-resistant. This means that the teeth in which there is altered root formation due to radiotherapy will still continue to erupt.

### 5.6 Radiation Caries

It is a rampant form of dental decay. It occurs in those individuals who receive a course of radiotherapy that includes exposure of the salivary glands.

It results from changes in the salivary glands and saliva, including [32]:

- Reduced flow
- Decreased pH
- Reduced buffering capacity
- Increased Viscosity
- Because of the reduced or absent cleansing action of normal saliva, debris accumulates quickly.

#### 5.6.1 Preventions [32]

- Daily application of topical 1% neutral sodium fluoride gel in custom-made applicator trays for 5 mins.
- Avoidance of dietary sucrose.
- Restorative dental procedures.
- Excellent oral hygiene.
- Extraction of those teeth which present with deep caries or are periodontally compromised.

### 5.7 Bone

Radiation therapy with therapeutic doses may lead to damaging effects on the bone of the maxillofacial region owing to damage to the vasculature of the periosteum and cortical bone.

It may also be due to the destruction of osteoblasts as well as osteoclasts. Post radiation, the normal marrow will be replaced by a marrow which will be fatty and with fibrous connective tissue [32].

Resultantly in a nut shell the marrow tissue becomes Hypovascular, Hypoxic and Hypocellular.Endosteum becomes atrophic, showing a lack of osteoblastic and osteoclastic activity. Some lacunae of the compact bone are empty which is an indication of necrosis. Degree of mineralization may be reduced, leading to brittleness. When bone death occurs because of these changes, the condition is termed osteoradionecrosis [34].

Osteoradionecrosis refers to an inflammatory condition of bone (osteomyelitis) that occurs after the bone bas been exposed to therapeutic doses of radiation usually given for a malignancy of the head and neck region. It is characterized by the presence of exposed bone for a period of at least 3 months occurring at any time after the delivery of the radiation therapy. This infection may result in Non healing wound in bone which is difficult to treat.

It is more common in the mandible than in maxilla because [32]

- Of the richer vascular supply to the maxilla.
- Mandible is more frequently irradiated.

The higher the radiation dose absorbed by the bone, the greater the risk for osteoradionecrosis.

#### 5.7.1 Precautions [32-37]

- Patients must be referred for dental care before undergoing a course of radiation therapy to reduce the severity of or prevent radiation caries and osteoradionecrosis.
- Extraction of all the periodontally compromised teeth. If the patient has undergone an extraction, sufficient time must be given for the extraction wound to heal before the initiation of radiotherapy.
- Atraumatic surgical technique
- Antibiotic coverage
- Low concentration epinephrine-containing local anaesthetics that do not contain lidocaine.
- Avoid taking radiographs during the 1^st^ 6 months after completion of radiotherapy to allow time for the mucosal membrane to heal

## RECOMMENDATIONS AND CONCLUSION

Training of radiation safety principles at an undergraduate level should be advocated. Continuing Dental Education (CDE) programmes must be made mandatory for the practicing dental professionals so as to impart them with current knowledge of diagnostic radiology and the risks associated with the same. The information regarding these CDE programmes must also be made available on social media as well as social websites so as to target a mass population of dentists. All the radiological equipment must be registered and Quality Assurence of the machine must be made mandatory before making the machine functional. It also involves periodic check-up of X-ray units for any radiation leak and for optimal parameters. This must be made mandatory for the renewal of registration. All the recommended guidelines must also be provided to the dentists. The dentists must also be educated to wear film badges of Thermolumnescent Dosimeters (TLD) for personal dosimetry. These should be made compulsory to be worn by the dentist and the para-medical staff as well. The dentists must also be instructed to periodically evaluate the quality of their X-ray images so as to ensure optimal exposure reduction as early as possible.
